# Editorial: Subclinical thyroid disease: present knowledge and future direction, volume II

**DOI:** 10.3389/fendo.2023.1293910

**Published:** 2023-10-09

**Authors:** Jose De Jesus Garduno Garcia, Alberto O. Chavez, Daniel Elías-López

**Affiliations:** ^1^Medicine school, Universidad Autónoma del Estado de México, Toluca, Mexico; ^2^General Hospital, Instituto Mexicano del Seguro Social (IMSS), Toluca, Mexico; ^3^Texas Diabetes Institute - University Health, UT Health San Antonio, San Antonio, TX, United States; ^4^Endocrinology Department, Instituto Nacional de Ciencias Médicas y Nutrición Salvador Zubirán (INCMNSZ), Mexico City, Mexico

**Keywords:** subclinical hypothyroidism, subclinical thyroid disease, hypothyroidism, cardiovascular risk factor, thyroid disease

Thyroid diseases are a major focal point in general endocrinology practice. The diversity within this spectrum poses a daily challenge in patient care (1). While the presentation of thyroid dysfunction – both hypo and hyperthyroidism – are extensively reviewed in the literature, the realm of subclinical thyroid dysfunction remains a diagnostic and treatment conundrum (2). This is further complicated by the occurrence of these issues across all stages of life, often coexisting and associating with prevalent diseases that significantly impact global public health (3). Recognizing the importance of this subject, a second volume on the topic is being undertaken, following the success of the initial one.

Cardiovascular diseases are the leading cause of mortality worldwide (4). This increase in cardiovascular risk is closely associated to the high prevalence of obesity and insulin resistance in the general population (5). In this regard, Yang et al. describes the relationship between insulin resistance with subclinical thyroid dysfunction in individuals with both normal glucose tolerance and diabetes. Similarly, Song et al. explores the existing relationship between various obesity phenotypes and overall thyroid function status.

For years, the initial assessment of thyroid function has predominantly revolved around measuring TSH as the primary determinant. Research on the significance of measuring thyroid hormones concentration in different contexts has been somewhat limited (6). Interestingly, the concentration of thyroid hormones might serve as markers for predicting outcomes in specific scenarios (7). This Research Topic explores this association in mortality in critically ill patients as well as in patients with fulminant myocarditis. Furthermore, the association between markers of thyroid function and cardiometabolic risk factors in children is also explored.

The impact of thyroid function extends also to factors such as aging (8), mental illness (9), and rare disorders. As such, an ongoing debate in endocrinology pertains to how aging affects hormonal function. Zhang et al. article explores thyroid function and its association with autoimmune phenomena in a population of older adults. Regarding mental health and thyroid dysfunction, Peng et al. presents an interesting analysis on the association of TSH with metabolic alterations in patients with major depression and a history of suicide attempts. Finally, is worth noting that a high prevalence thyroid dysfunction can coexist in certain rare diseases such as the case of mesenchymal tumors of the thyroid gland. Zhang and Liu carries out an interesting review of these tumors and explores their pathophysiology.

The papers collected in this second volume substantially contribute to our overall understanding of subclinical thyroid disease. Nevertheless, numerous questions remain unanswered, highlighting the need for further research to elucidate the intricate mechanisms behind these conditions and their broader implications for health.

## Author contributions

JG: Writing – original draft. AC: Writing – review & editing. DE: Writing – review & editing.
